# Utility of the evaluation of blood flow of remnant esophagus with indocyanine green in esophagectomy with jejunum reconstruction: Case series

**DOI:** 10.1016/j.amsu.2020.12.008

**Published:** 2020-12-05

**Authors:** Kenjiro Ishii, Yasuhiro Tsubosa, Junichi Nakao, Ryoma Haneda, Yoshitaka Ishii, Eisuke Booka, Shuhei Mayanagi, Jun Araki, Yoshichika Yasunaga, Masahiro Nakagawa

**Affiliations:** a Division of Esophageal Surgery, Shizuoka Cancer Center, 1007 Shimonagakubo, Nagaizumi-cho, Suntou-gun, Shizuoka, 411-8777, Japan; b Division of Plastic and Reconstructive Surgery, Shizuoka Cancer Center, 1007 Shimonagakubo, Nagaizumi-cho, Suntou-gun, Shizuoka, 411-8777, Japan; c Department of Plastic Reconstructive Surgery, Hamamatsu University School of Medicine, 1-20-1 Hanndayama, Higashi-ku, Hamamatsushi, Shizuoka, 431-3192, Japan; d Department of Surgery, Hamamatsu University School of Medicine, 1-20-1 Hanndayama, Higashi-ku, Hamamatsushi, Shizuoka, 431-3192, Japan

**Keywords:** Esophageal reconstructive surgery, Indocyanine green, Remnant esophagus, Esophagectomy, Microvascular anastomosis

## Abstract

**Background:**

Pedicled jejunal flap can be utilized with various tips for esophageal reconstruction in patients with a history of gastrectomy or those who have undergone synchronous esophagogastrectomy. However, the rate of anastomosis leakage is high; therefore, we considered the evaluation of blood flow of the remnant esophagus with indocyanine green in setting the anastomosis site.

**Methods:**

Fifty patients who underwent radical esophagectomy with pedicled jejunal flap between January 2011 and June 2020 were identified. From June 2019, blood flow in the pedicled jejunum and remnant esophagus were evaluated to set the anastomosis site of the latter. Usually, the second and third jejunal vessels are transected, and if the jejunal flap cannot reach to the anastomosis point, we actively transect the marginal vessels to stretch the jejunal flap. Microvascular anastomosis between the jejunal branches and the internal thoracic vessels is usually made, and the anastomosis site is set at the well-stained part of the esophagus.

**Results:**

Overall, 39 patients underwent the procedure before June 2019 (Group A), and 11 patients underwent the procedure since June 2019 (Group B). No significant difference was found in the patients’ background, type of preoperative therapy, presence or absence of ligation of marginal vessels and two-stage operation between the groups. Group A had 16 cases of anastomosis leakage; B had only 1 case (*p* < 0.05). There were no cases of pedicled jejunum graft necrosis.

**Conclusion:**

Assessing remnant esophageal perfusion by indocyanine green imaging in pedicled jejunum reconstruction resulted in a lower anastomotic leak rate.

## Highlights

• ・Pedicled jejunal flap can be utilized with various tips for esophageal reconstruction, although the rate of anastomosis leakage is high.
• ・we considered the utility of the evaluation of blood flow of the remnant esophagus with indocyanine green in setting the anastomosis site.
• ・The anastomosis site is set at the part of the esophagus that is well-dyed with indocyanine green.
• ・Our findings demonstrate that the evaluation of the blood flow of the remnant esophagus during setting of the anastomosis site may decrease the rate of anastomosis.

## 1 Introduction

The stomach is generally used for esophagectomy reconstruction, however, synchronous occurrence of gastric cancer or metastatic gastric lesions are frequently detected in esophageal cancer patients. Indeed, it has been reported that synchronous occurrence of gastric cancer is observed in 4.3% of esophageal cancer patients in 2007 [1]. Moreover, 3.1% of the esophageal cancer patients in the current study had a history of treatment for gastric cancer prior to surgery for esophageal cancer. In addition, patients with severe gastric ulcer and gastric ulcer perforation also require gastrectomy until the development of effective proton pump inhibitors.

There are two choices for esophageal reconstruction in patients after gastrectomy or in those who undergo synchronous esophagogastrectomy, pedicled jejunum or colon graft interposition. No previous reports have found significant differences between the utility of these two procedures; however, in Japan, the use of the pedicled jejunal graft has been gradually increasing [[2], [3], [4], [5], [6], [7]].

Advantages of the pedicled jejunum include fewer anastomoses, vigorous peristalsis, and the fact that malignancies are rare in the jejunum [8]. However, the length of the jejunal graft is restricted by mesenteric arcade. In order to achieve sufficient length for reconstruction, the second and third mesenteric branches need to be ligated, and the vessel arcade is sometimes ligated too. Therefore, microvascular anastomosis between the jejunal branches and the internal thoracic or cervical vessels is frequently necessary in order to generate sufficient blood flow. Furthermore, there is no reservoir in the pedicled jejunum, and there is no randomized controlled study comparing the outcomes of pedicled jejunum and colon graft interposition; however, the mortality rates seem to be higher in patients who undergo colon graft interposition compared to those who undergo pedicled jejunal graft [8].

It is generally accepted that the main factors that require consideration in the anastomosis of the esophagus to the jejunum or gastric tube are the blood flow and the tension of the anastomosis [[9], [10], [11]]. The indication for microvascular anastomosis likely depends on the institutes or the surgeons, and therefore, few reports recommend adding the vascular anastomosis. Currently, several researches have investigated the usefulness of indocyanine green fluorescence (ICG) imaging for the assessment of blood perfusion of the gastrointestinal tract during surgery after intravenous injection, including gastric conduit and free jejunal graft surgery [[12], [13], [14], [15], [16]].The flow of ICG can be visualized with a near-infrared camera, and real-time information of the blood supply can be acquired. However, the usefulness of ICG imaging for pedicled jejunum graft and remnant esophagus has not been adequately studied.

In pedicled jejunum reconstruction, the elongated jejunal graft is commonly pulled-up through the subcutaneous or retrosternal route [8]. The jejunal graft has insufficient length as it is restricted by mesenteric arcade; therefore, the remnant esophagus tends to be slightly longer than in the gastric conduit, especially when the subcutaneous route is used. In line with this, we consider that the blood supply of the remnant esophagus contribute to the esophagojejunal anastomosis and it is also important to evaluate the blood flow of the edge of the remnant esophagus, which is used for the anastomosis, in order to determine its utility.

In this report, we assessed the utility of ICG fluorescence imaging for evaluation of the perfusion of the remnant esophagus during esophagectomy with pedicled jejunum reconstruction through the subcutaneous route.

## 2 Material and methods

### 2.1 Study design and surgical technique

Between January 2011 and June 2020, 50 patients underwent esophagectomy with pedicled jejunum reconstruction at our institute. The patients underwent subtotal esophagectomy via right thoracotomy with either an endoscopic or open procedure. Mid-upper abdominal open laparotomy was performed, and a pedicled jejunum and jejunostomy were made. The first branch of the superior mesenteric vessels are preserved for perfusion to the distal duodenum, and, in all cases, the second mesenteric branch is dissected and divided to supercharge the proximal flap. The third mesenteric branch is also divided in almost all cases. If the jejunum graft was still unable to reach, the vessel arcade between the second and third vessels was divided in order to secure additional length of the graft. Reconstructions were performed through the subcutaneous route, with handsewn anastomosis at the ante thoracic or cervical location. Microvascular anastomosis between the jejunal branches (artery and vein, or only vein, at second mesenteric branch) and the internal thoracic vessels (second or third intercostal space) is usually made by plastic surgeons.

If patients are considered to be high-risk with regards to an invasive operation, such as those with older age (≥75 years or more), low respiratory function, and severe diabetes mellitus, two-stage operations can be performed, in which reconstruction is usually performed approximately 3 weeks after the first operation.

We evaluated the blood flow of both the pedicled jejunum and the remnant esophagus with the use of ICG imaging from June 2019. We divided the patients into two groups: Group A (n = 39; no evaluation of remnant esophagus with ICG imaging) and group B (n = 11; using ICG imaging for the evaluation of remnant esophagus). We retrospectively investigated the patients’ baseline characteristics and preoperative therapies, as well as factors related to the reconstruction procedure (interruption of mesentery arcade, microvascular anastomosis, two-stage operation), and leakage of esophagoiejunal anastomosis.

### 2.2 Evaluation of the blood flow with the use of ICG imaging

**Fig. 1:**
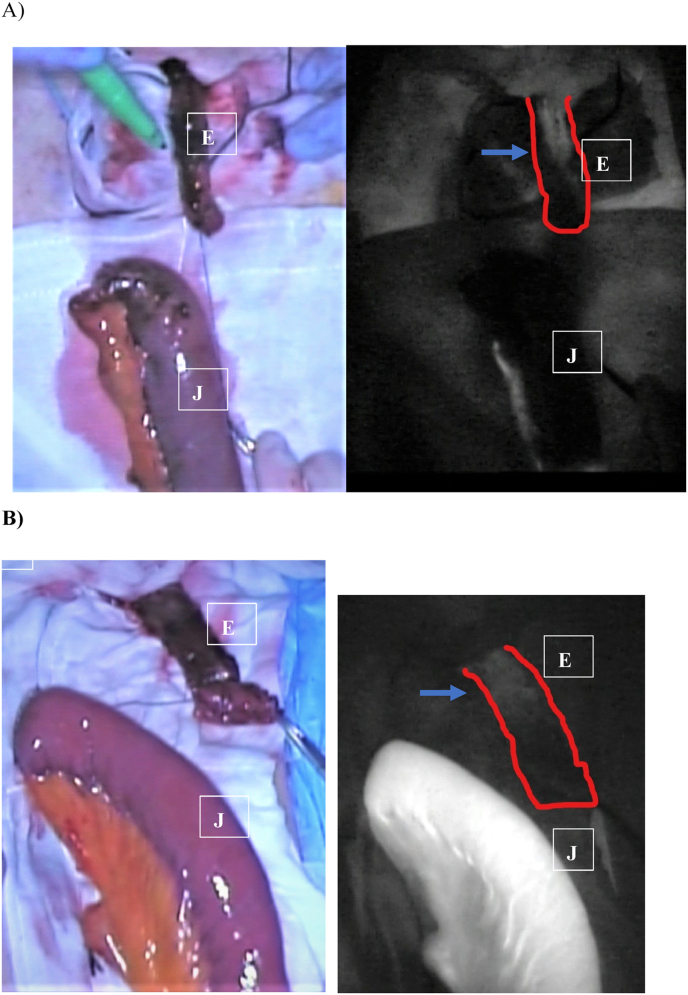
ICG imaging of remnant esophagus and pedicled jejunumLeft pictures demonstrate the remnant Esophagus E), and pedicled jejunum J). The mesentery arcade was divided in A), and preserved in B).Right pictures demonstrate ICG imaging with black or white modeArrow pointing to the edge of the white dye area in ICG imaging, defined as the anastomosis site of the remnant esophagus.

Before microvascular anastomosis, ICG (1 mL, Diagnogreen 0.5%; Daiichi-Sankyo Pharmaceutical, Tokyo, Japan) was intravenously injected. ICG absorbs light in the near-infrared range, with a maximum wavelength of 805 nm. ICG fluoresces, with a maximum wavelength of 840 nm in plasma [17]. ICG fluorescence imaging was performed using a near-infrared camera system (HAMAMATS PHOTONICS K·K, Shizuoka, Japan), and the images were displayed on a monitor with black or white mode (dying white indicates blood perfusion). In cases where it was possible to preserve the mesentery arcade, if the color of the jejunum was remarkably good and blood perfusion was detected with ICG imaging, microvascular anastomosis was only performed in the vein. Otherwise, when the vessel arcade was divided between the second and third vessels, the microvascular anastomosis of both of artery and vein of second mesenteric branch was necessarily made. Subsequently, we set the cutting site of the remnant esophagus according to the extent of white dying in ICG imaging (Fig. 1). If the remnant esophagus was dyed well to the anal side edge, only the edge was cut. Prior to the use of ICG imaging, the remarkably off color site was removed, and we aimed to preserve the length of the remnant esophagus as best we could.

### 2.3 Postoperative assessment

The assessment of anastomotic healing was performed under fluoroscopy by oral intake of a diatrizoate meglumine 6th or 7th postoperative day, and if there was no leakage and remarkable stenosis, jelly diet was started the next day. If a leakage was assumed due to clinical signs, we performed an upper gastrointestinal endoscopy.

### 2.4 Statistical analysis

Data are reported as medians and the mean ± standard deviation. and statistical significance was considered with a *p*-value < 0.05. The univariate analysis data were tested using the Pearson's chi-square, Fisher's exact test, and Mann–Whitney *U* test.

All of the statistical analyses were performed using SPSS (version 26.0, IBM Corporation, NY, USA).

This research has been reported in line with the PROCESS criteria [18].

## 3 Results

### 3.1 Baseline characteristics

Group A comprised 37 men and 2 women (median age, 68; range, 42–77 years), while Group B comprised 11 men and 1 woman (median age, 67; range, 50–77 years) (Table 1). The preoperative body mass index in both groups were 21.7 ± 3.0 kg/m^2^ (Group A) and 20.9 ± 3.7 kg/m^2^ (Group B), there was no significant difference between the two groups. No significant differences were found in terms of performance status, diabetes mellitus, or history of smoking (within 1 year). Both groups of patients comprised several clinical Stages, although there was no significant difference between the two groups.

**Table 1:** Baseline characteristics.

	Group A (n = 39)	Group B (n = 11)	*p*-value
**Male:female ratio**	37/2	10/1	*n.s*
**Age**	68 (42–77)	67 (50–77)	*n.s*
**BMI (kg/m**^**2**^**)**	21.7 ± 3.0	20.9 ± 3.7	*n.s*
**Performance status**			*n.s*
0	31	7	
1	6	4	
2	2	0	
			
**Diabetes mellitus**	8	2	*n.s*
**Smoking**	7	4	*n.s*
**(within 1 year)**			

**Clinical tumor staging (UICC 8th)**			*n.s*
cStage Ⅰ	10	2	
cStage Ⅱ	7	2	
cStage Ⅲ	9	3	
cStage Ⅳ A	8	3	
cStage Ⅳ B	5	1	

There was no significant difference between groups.

*n.s*: Not significant difference, BMI: Body mass index.

### 3.2 Preoperative therapy

Group A included 14 patients with no therapy, 19 patients who received chemotherapy, and 6 patients who received chemoradiotherapy prior to surgery (Table 2). Group B had 2 patients with no therapy, 5 patients who received chemotherapy, and 4 patients who received chemoradiotherapy prior to surgery. There was no significant difference in preoperative therapy between the groups.

**Table 2:** Preoperative therapy.

Type of therapy before operation	Group A (n = 39)	Group B (n = 11)	*n.s*
No therapy	14	2	
Chemotherapy	19	5	
Chemoradiotherapy	6	4	

There was no significant difference between groups.

*n.s*: No significant difference.

### 3.3 Factors related to the reconstruction procedure

There was no significant difference in the presence or absence of interruption of mesentery arcade between the groups (Table 3). Moreover, no significant difference was also found with regards to the decision to make microvascular anastomosis of only the vein or both the artery and vein.

**Table 3:** Factors related to reconstruction procedure.

	Group A (n = 39)	Group B (n = 11)	*p*-value
**Interruption of mesentery arcade (yes/no)**	24/15	5/6	*n.s*
**Microvascular anastomosis**			*n.s*
Only vein	4	3	
Artery and vein	35	8	
**Two-stage operation (yes/no)**	20/19	5/6	*n.s*

There was no significant difference in factors between groups.

*n.s*: Not significant difference.

In cases where patients have several risk factors for invasive surgery, we occasionally perform a two-stage operation. No significant differences were found between the two groups with regards to the use of a two-stage operation.

### 3.4 Leakage and stenosis of esophagojejunal anastomosis

Group A had 16 cases of leakage of esophagojejunal anastomosis (41.0%), while Group B had only one case (9.1%, P < 0.05) (Table 4).

**Table 4:** Leakage and stenosis of esophagojejunal anastomosis.

	Group A (n = 39)	Group B (n = 11)	*p-value*
**Anastomotic leakage (yes/no)**	16/23	1/10	*< 0.05*
**Anastomotic stenosis (yes/no)**	4/35	2/9	*n.s*

There was a significant difference in the anastomotic leakage between groups.

*n.s*: Not significant difference.

None of the cases required reoperation due to necrosis of the pedicled jejunum graft. Furthermore, all leakages could be controlled by opening the chest incision and drainage.

Only one case of leakage in Group B was detected on 17th postoperative day, when the patient had already been discharged; this case had interruption of the mesentery arcade, microvascular anastomosis of the vein and artery of the jejunal graft, and underwent a two-stage operation. This leakage could be controlled by opening the incision near the anastomosis and drainage. The color of the remnant esophagus and pedicled jejunum was good, and the cause of leakage was not considered to be ischemia.

With regards to the stenosis of the esophagojejunal anastomosis that required endoscopic dilatation, there were four cases in Group A and two cases in Group B, and there was no significant difference between the two groups.

## 4 Discussion

The rate of anastomotic leakage in pedicled jejunum reconstruction was higher than that in the gastric conduit, and the rate was reported to be from 7.4% to 36.4% [[18], [19], [20], [21], [22], [23], [24], [25], [26]]. In this report, the rate in Group A was 41.0%, which was relatively high. However, there were no cases of graft necrosis in our study, which is reported to range from 0% to 7.7% [[19], [20], [21], [22], [23], [24], [25], [26], [27]]. This observation indicates that microvascular anastomosis of jejunal vessels was clearly effective for blood perfusion to pedicled jejunum graft, therefore we focused on the blood supply of the remnant esophagus, although there was no clear evidence about it.

Several factors are related to good anastomosis between the esophagus and the jejunum graft, including blood perfusion, tension, and patient's background. Furthermore, there was no significant difference in the patients' background and factors with regards to the reconstruction procedure of the pedicled jejunum between two groups; however, the rate of anastomotic leakage was considerably decreased in Group B. Although small in number, this may imply that the blood supply to the cut end of the remnant esophagus is an important factor in esophagojejunal anastomosis.

As noted above, one of the disadvantages of pedicled jejunum reconstruction is the restricted length of the jejunal graft. In the current study, we divided the vessel arcade in an affirmative way in order to acquire a sufficient length of the graft. However, we still waited for as long as possible to secure the length of the remnant esophagus for anastomosis, so as to reduce the tension to the anastomosis.

In terms of blood flow, in the neck, esophageal blood flow is supplied by multiple small branches from the inferior thyroid artery, and the inferior laryngeal branch of the inferior thyroid artery also anastomoses with the superior laryngeal artery from the superior thyroid artery. While in the thorax, 4–5 esophageal arteries arise directly from the aorta. Moreover, 1–2 esophageal branches arise from bronchial arteries, and sometimes from an intercostal artery, and there is a fine esophageal intra mural blood flow [[28], [29], [30], [31]]. Although we check the color of remnant esophagus, the color is not definitive criteria for judging in surgery on the remnant esophagus, whereas the evaluation of blood supply in pedicled jejunal graft can be relatively easy with the color and peristaltic motion. In many cases, esophageal branch from the inferior thyroid artery is divided in esophagectomy, and the blood supply in the remnant esophagus can be maintained to the cut end of the upper esophagus by only intra mural blood flow from the cervical esophagus. Therefore, the blood supply can be poor near the cut end. Even though the color is good, sometimes poor dyeing in ICG imaging is found near the cut end. In that case, we should divide the mesojejunum vessel arcade (between the second and third mesenteric branch) to achieve the long enough jejunal graft, cut the well dyed area of remnant esophagus, and have a plastic surgeon perform microvascular anastomosis because poor blood flow at the remnant esophagus could lead to anastomosis leakage.

There are some limitations to the current study. First, our analysis was retrospective and conducted in a single center limited to an Asian population. Also, we have extensive experience and workload in esophageal cancer surgery due to a high local incidence, and as such, our outcomes may not be applicable to centers in other countries. Moreover, our sample size was small, and the evaluation of ICG imaging is subjective; therefore, there is a possibility of bias in the interpretation.

## 5 Conclusion

Our series showed that assessing remnant esophagus perfusion assessment by ICG imaging during esophageal reconstruction with pedicled jejunum resulted in a lower anastomotic leakage rate compared to historical controls. The key message is that careful attention to the blood flow of not only jejunal graft but also remnant esophagus in esophagectomy with pedicled jejunum reconstruction.

A prospective trial with a large number of patients with pedicled jejunum reconstruction is necessary in order to standardize the use of ICG imaging.

## Ethical approval

The protocol was reviewed and approved by the local ethics committee.

## Sources of funding

This research did not receive any specific grant from funding agencies in the public, commercial, or not-for-profit sectors.

## Provenance and peer review

Not commissioned, externally peer reviewed.

## Author contribution

Please specify the contribution of each author to the paper, e.g. study concept or design, data collection, data analysis or interpretation, writing the paper, others, who have contributed in other ways should be listed as contributors.

Study conception and design: K Ishii, Y Tsubosa, J Nakao.

Acquisition of data: K Ishii, E Booka, R Haneda.

Analysis and interpretation of data: K Ishii, J Nakao, Y Tsubosa.

Drafting of manuscript: K Ishii, E Booka, Y Tsubosa, Y Ishii, J Nakao, J Araki, Y Yasunaga, M Nakagawa.

Critical revision: K Ishii, Y Tsubosa, J Nakao, Y Yasunaga, M Nakagawa.

## Declaration of competing interest

The authors declare that they have no conflicts of interest.
